# Personal

**Published:** 1900-05

**Authors:** 


### PERSONAL.

Dr. Francis E. Fronczak, of 508 Fillmore Avenue, Buffalo, was married, April 25, 1900, to Miss Lucy Rosalie Tucholka, likewise of Buffalo. After a reception Dr. and Mrs. Fronczak left for New York and on April 28th, sailed for Gibraltar and a short tour through the Mediterranean, expecting to visit several African ports. They will also visit Italy, Switzerland, the Oberammergau Passion Play, and on June 5-7th will take part in the jubilee of the 500th anniversary of the foundation of the Jagellenian University at Cracow, Austria, to which Dr. Fronczak will serve as a delegate from the University of Buffalo. He will study at the clinics of the universities of Cracow and Warsaw, will take part in the ninth congress of Polish Physicians and Scientists, which will be held from July 21-25, at Cracow, where he will represent the American Medical Association, and deliver one of the four public addresses. The other three speakers, one each day, will be Professor Neucki, of the University of St. Petersburg; Prof. Mikulicz, a third some Polish celebrity from Switzerland. Dr. Fronczak's subject will be The Progress and Present Status of Medicine in America.

From Cracow Dr. Fronczak will leave with the Polish contingent for Paris, where they will take part in the thirteenth International Medical Congress. Dr. Fronczak is registered in the medical jurisprudence section of the congress. Dr. Fronczak and wife, and Mr. Speyszynski, a Polish druggist from East Buffalo, who will accompany them, will return to Buffalo about August 25th.

---

Dr. Charles F. Howard, of Buffalo, has been appointed by Governor Roosevelt to be a member of the board of managers of the Elmira Reformatory. This came somewhat in the nature of a surprise, for it was not generally known or even suspected that a Buffalonian would be chosen.

Dr. Howard will go into the board thoroughly equipped to remedy evils that are believed to exist and assist in instituting reforms in internal management, which for years have been needed, according to the newspapers.

It will be remembered that the first public attack on so-called Brockwayism was made in Buffalo by the *Evening News*, which was the prelude to the general newspaper fight against the methods employed at the reformatory by newspapers in this state.

Dr. Howard's sympathies, it appears, were aroused by the reports of abuse and ill-treatment which came to Buffalo at that time; and ever since then he has taken an active interest in the affairs of what should be a model institution. He has studied the questions involved in the management of the institution and by his appointment it appears to the JOURNAL that a man is chosen as manager who is peculiarly well fitted to fill with judgment and intelligence, a place which has been made vacant by resignation of one of the supporters of the existing régime.

Dr. Howard is a graduate of the University of Buffalo, class of '78, and has been practising in this city for more than twenty years.

---

DR. EDWARD CHAPIN, of Brooklyn, has been appointed by the regents of the University of the State of New York, a member of the Homeopathic Board of State Medical Examiners, to fill the vacancy caused by the death of Dr. A. R. Wright, of Buffalo.

---

DR. EUGENE WASDIN, Surgeon U. S. Marine Hospital Service, lately ordered to Buffalo to relieve Passed Assistant Surgeon J. B. Stoner, has arrived at his post and assumed command of the service at this station. Dr. Stoner has been ordered to St. John, N.B.

